# Neuro-ophthalmic complications of immune checkpoint inhibitor therapy: Current status and future directions

**DOI:** 10.3389/fopht.2022.1044904

**Published:** 2022-11-18

**Authors:** Kimberly M. Winges, Lynn K. Gordon

**Affiliations:** ^1^ Casey Eye Institute Division of Neuro-Ophthalmology, Oregon Health & Science University School of Medicine, Portland, OR, United States; ^2^ Veterans Affairs Portland Health Care System, Ophthalmology Department /Operative Care Division, Veterans Health Administration, Portland, OR, United States; ^3^ Jules Stein Eye Institute Division of Neuro-Ophthalmology, University of California Los Angeles David Geffen School of Medicine, Los Angeles, CA, United States

**Keywords:** immune checkpoint inhibitor, neuro-ophthalmology, CTLA-4, PD-1, PD-L1, immune-related adverse events, pembrolizumab, melanoma

## Abstract

Since 2011, use of immune checkpoint inhibitors (ICI) in cancer immunotherapy dramatically expanded, both alone and in combination with either a different cancer treatment or with two different ICIs. With this increase in use have come a myriad of adverse effects from enhanced immune activation, including ophthalmic and neurologic immune related adverse events (irAE). Neuro-ophthalmic immune related adverse events (NOirAE) associated with use of ICIs are increasingly recognized and their severity may actually limit use of potentially life-saving immunotherapy. NOirAEs comprise a wide variety of presentations involving both the central and peripheral nervous system. They cause afferent or efferent visual dysfunction, including among them optic neuropathy and edema, orbital inflammatory disease, and ocular myasthenia. While treatment for irAEs typically involves immunosuppression with corticosteroids, there is no expert consensus regarding best practices for treatment of NOirAEs and whether to stop ICI immunotherapy for the cancer or not. This state-of-the-art review explores the pathophysiologic basis for NOirAEs, provides a framework for categorizing them within neuro-ophthalmology, and discusses what is needed to close the current knowledge gaps in diagnosis and management of an increasing population of cancer patients requiring neuro-ophthalmic care.

## Introduction

The burgeoning field of targeted cancer therapeutics has increasingly taken advantage of recent discoveries in the field of immunology, facilitating the development of efficacious molecular therapies to decrease specific points in the cancer immune response cascade. Perhaps the most exploited is the T cell activation cascade, and knowledge about specific cytotoxic CD8+ T cell mechanisms has given modern medicine multiple therapeutic targets to block, and therefore modify, the immune response to cancer cells. As a result, immuno-oncology research has created a sea change in the approach to fighting cancer, moving from directly killing tumor cells to enhancing the host’s own immune response to fight off malignancy. Favorable survival rates and durability of effect have propelled these treatments into first line treatment above chemotherapy in some malignancies, starting with melanoma and non-small cell lung cancer (NSCLC) (1). Due to their unique mechanism, however, side effects of these treatments are immunogenic rather than cytotoxic, and they affect almost every organ system. This review summarizes what is known and what knowledge is needed regarding ICI immune-related adverse events (irAE) for the neuro-ophthalmologist, who must address both ophthalmic and neurologic side effects of immune reactivation.

## Immune basis for ICI therapy

**Figure 1:**
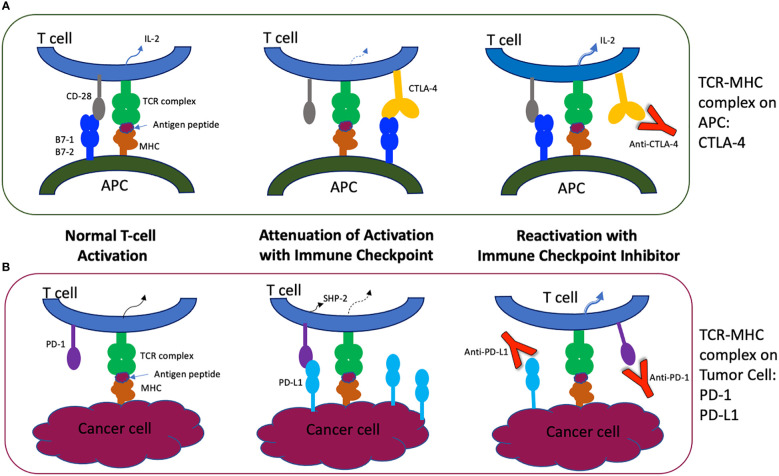
Molecular Mechanisms of Immune Checkpoints and Their Inhibitors. **(A)** The primary activation of the naïve T cell derives from the T cell receptor/CD3 Complex interacting with the antigen presenting cell’s MHC Complex, which presents the tumor antigen peptide and stimulates a cytokine cascade within the T cell. CTLA-4 competitively binds to the co-stimulatory molecule CD28, attenuating the T cell activation and providing co-inhibition of the immune response in normal cells. Antibodies targeting CTLA-4 inhibit the co-inhibition pathway, resulting in a more robust and sometimes inappropriate immune activation mediated by unopposed T cell activities. **(B)** T cells also interact with other APCs and cancer cells through the co-inhibitory interaction of PD-1 with its ligands, PD-L1 and PD-L2 (not shown). Tumor cells may overexpress PD-L1 and lead to over-attenuation of the T cell response *via* the SHP-2 pathway. Antibodies to PD-1 and PD-L1 restore T cell activation, allowing T cell differentiation and cytokine production but also inflammatory side effects (2, 3). CD3, complement domain 3. MHC, major histocompatibility complex. CTLA-4, cytotoxic T lymphocyte associated protein 4. PD-1, programmed cell death protein 1. PD-L1, programmed cell death ligand-1. APC, antigen presenting cell. SHP-2, phosphatase Src homology 2 domain-containing phosphatase-2. Adapted from (2).

T cell activation requires a two-signal process that utilizes the T cell receptor (TCR) paired with a peptide presented by its antigen presenting cell (APC, an MHC Class I or II molecule), as well as a co-stimulatory molecule interaction with its APC ligand. This interaction then activates the otherwise anergic T cell to produce appropriate cytokines through intracellular signaling pathways. When these cascades are initiated, co-inhibitory receptors are also activated to prevent overstimulation of the immune system by the antigen (see **Figure 1**).

One such co-inhibitory pathway involves the cytotoxic T-lymphocyte associated protein 4 (CTLA-4) on the T cell membrane. By competitive binding with its co-stimulatory molecule CD28 to ligands B7-1 (CD80) and B7-2 (CD86) on the APC, CTLA-4 limits T cell proliferation and the formation of cytokine IL-2, protecting the body from an overzealous immune response (4–6).

Another T cell membrane co-inhibitory pathway is mediated by PD-1 (programmed cell death protein 1, CD279), an immunoglobulin superfamily protein that binds to programmed cell death ligand-1 and -2 (PD-L1 and PD-L2). PD-L1 is widely expressed and when bound to PD-1 on the T cell, the phosphatase Src homology 2 domain-containing phosphatase-2 (SHP-2) inhibits key kinases of the proximal TCR signaling molecules and leads to suppression of T cell activation (5). The PD-1/PD-L1 pathway also addresses T cell exhaustion and tolerance (1, 7).

In many tumor microenvironments, tumor cell expression or overexpression of PD-L1 binds circulating T cells through PD-1, leading to inactivation of potentially cytotoxic T cells. Monoclonal antibodies to CTLA-4 and PD-1 or PD-L1 therefore remove the inhibition and allow the body to enhance its immune response to the cancer through restoring cytotoxic T cell activation (5, 6, 8).

Currently approved anti-CTLA-4 agents include the monoclonal antibody ipilimumab and tremelimumab. The anti-PD-1 monoclonal antibodies are pembrolizumab, nivolumab, and cemiplimab. Anti-PD-L1 monoclonal antibodies are atezolizumab, avelumab, and durvalumab. While FDA-approved for use in various solid and hematologic malignancies, most commonly cutaneous melanoma and non-small cell lung cancer (9), efficacy is not universal and ICI-ICI combination therapy (anti-CTLA-4 plus anti-PD-1) as well as ICI-chemotherapy or ICI-radiation treatment have emerged as potential solutions (10).

## ICI side effects

Unfortunately, when normal co-inhibitory mechanisms are blocked with ICI therapeutics, T cell cascades have the potential to become overly active, leading to a variety of systemic inflammatory responses over and above the desired therapeutic effect. Immune-related adverse events (irAEs) are identified in almost every organ, involving skin, gastrointestinal, endocrine, hepatic, neurologic, ocular, and pulmonary systems. Compared with conventional chemotherapy, overall safety studies show that ICIs cause rash, diarrhea, colitis, pruritis, thyroid toxicity, and pneumonitis; two ICI drugs, or one in combination with chemotherapy, are associated with hepatic toxicity (11–16). IrAEs are coded in severity from grades 1-4, the highest grade being most severe and potentially life-threatening. Grade 1 is usually managed in the outpatient setting without cessation of drug, while grade 2 through 4 may require stopping the ICI, glucocorticoid administration, or other immunosuppressants. Grades 3 and 4 are usually managed through an inpatient setting (see **Table 1**) (18).

**Table 1:** Grading of immune-related adverse events with an example relevant to the neuro-ophthalmologist (17).

Grade		Example
**Grade 1**	**Mild symptoms or asymptomatic:** clinical diagnostic observation, requiring only conservative, non-systemic intervention.	Dry eye syndrome
**Grade 2**	**Moderate:** minimal, local, limiting instrumental activities of daily living, may need low-dose systemic steroid, may be able to continue ICI after treating with return to Grade 1.	Mild disc edema without visual field defects
**Grade 3**	**Severe but not immediately life-threatening:** hospitalization or prolongation of existing hospitalization, limiting self-care activities of daily living, high dose steroid and/or other immunosuppressants, ICI cessation.	Orbital myositis with optic nerve compression
**Grade 4**	**Life-threatening:** acute care hospitalization or transfer to acute care during existing hospitalization, high dose steroid and/or other immunosuppressants, permanent ICI cessation.	Immune-related myasthenia gravis causing respiratory distress

In a large meta-analysis of 36 phase II/III clinical trials looking at 5 ICIs, overall systemic safety profiles ranked from least to most irAEs were: Atezolizumab, nivolumab, pembrolizumab, ipilimumab, and tremelimumab (19). Integrated evidence based on pooled data, including correcting for differing study dosing regimens, incidence, subgroup of cancer type and type of irAE, suggests that nivolumab was overall the safest drug, especially in lung cancer (19). However, this statement is a moving target, as increasing post-approval retrospective analyses occur with new and combination therapies. Despite the safety concerns, patients on ICI drugs have superior survival and efficacy compared to traditional chemotherapy in some types of malignancies, and they therefore have moved into first line treatment for select cancers such as lung and melanoma (1). The oncologist must thus trade their expertise in management of traditional cytotoxic side effects, such as nausea, anemia, and immunosuppression, for growing expertise in managing endocrinopathies, pneumonitis, neuropathies, and hepatitis. This shift has made neuro-ophthalmic side effects much more prevalent in cancer treatment, and there is a need both for oncologists and neuro-ophthalmologists to understand the epidemiology, diagnosis, and management of NOirAEs in this new patient population.

## Ophthalmic immune-related adverse events

As would be expected, ophthalmic immune-related adverse events (OirAEs) are primarily inflammatory, and much of the data comes from large retrospective databases or small case reports. Ophthalmic side effects are often reported as less than 1% (4, 20, 21). Nevertheless, a recent review of nearly 1000 patients from the Mayo Clinic reported 2.8% ophthalmic side effects, most commonly dry eye, inflammatory uveitis, and myasthenia gravis (22). A federal adverse event reporting system (FAERS) study, focused on post-consumer reporting of spontaneous adverse events after ICI administration, looked at a disproportionality analysis of ICI OirAE reporting to FDA from 2003-2018 and concluded that atezolizumab had the highest association with eye inflammation (encompassing all types of ocular inflammation “including uveitis, endophthalmitis, and other ocular inflammatory diseases”), and ipilimumab had the highest association with uveitis specifically (23). A retrospective study of the Intelligent Research in Sight (IRIS) Registry from the American Academy of Ophthalmology identified 112 patients with OirAEs (24). Incidence rates were highest for anterior uveitis, and a prior diagnosis of ocular inflammation increased high risk of recurrence while on ICI therapy. Finally, a retrospective Kaiser Permanente database search in Southern California revealed a higher 1-year incidence of uveitis in patients on ICIs who had a diagnosis of melanoma (1.2% overall, largely driven by CTLA-4 targets) over non-melanoma cancer (0.2%, Odds Ratio of 6.45) (21). Non-uveitic ophthalmic complications (scleritis, papilledema, optic neuritis, optic atrophy, cranial neuropathies 3,4,6, internuclear ophthalmoplegia, or myasthenia gravis) showed no 1-year incidence difference for patients with melanoma or patients with non-melanoma cancers. This study found especially high 1-year recurrence rates of uveitis in patients with a history of uveitis, regardless of cancer type. Taken together, these research studies have made retrospective observations with varying degrees of epidemiologic and population data, and they are difficult to assemble into conclusive recommendations due to varying inclusion criteria and methodology.

Regarding treatment, patients are managed largely by traditional investigations to rule out mimickers of inflammation (such as metastasis to vitreous), treatment with varying doses of corticosteroids, and when necessary and in concert with the oncologist, by stopping the ICI treatment. However, these retrospective database inquiries vary by size, frequency, severity, treatment selection, and recovery. Currently there are no consensus statements on diagnosis and management of ophthalmic irAEs.

## Neurologic immune-related sequelae

Neurologic immune-related sequelae of ICIs occur in 1-12% of patients, especially those with underlying neurologic disease or combination therapy (25–28). They include headache, encephalopathy, myasthenia gravis, neuropathies, and myositis, among others (29). They frequently cause diagnostic uncertainty due to vague symptoms with underlying malignancy, which can be causally related. Peripheral nervous system manifestations are twice as frequent as those within the central nervous system. Given their generally higher severity than non-neurologic ICIs, as well as their increased frequency compared to neurologic sequelae of other cancer treatments, the oncology and neurology communities have attempted to provide formal recommendations for diagnosis and management of neurologic irAEs based on current evidence (25–32). According to a recent Delphi consensus group report, the neurologic irAEs are organized into 7 “core syndromes”: meningitis, encephalitis, demyelinating disease, vasculitis, neuropathy, neuromuscular junction disorders, and myopathy (33). In this classification scheme, the core syndromes each contain syndrome subtypes, ie. optic neuritis as the subtype of a demyelinating syndrome. A naming schema is then recommended and based on diagnostic certainty, severity, autoantibody association, exacerbation of pre-existing disease or *de novo* presentation, and presence or absence of concurrent irAE(s) (33).

Treatment of neurologic irAEs relies as well on prior experience with non-ICI associated disease therapy and depends on severity of neurologic symptoms. Most neurologic sequelae recover completely with steroids, but some require targeted treatment such as intravenous immunoglobulin (IVIG), gabapentin, or anti-hypertensives (32). Treatment choice varies by etiology and anatomic location, but the European Society for Medical Oncology (ESMO) practice guidelines recommend holding the ICI therapy and performing a work-up (MRI scan, lumbar puncture, serum testing) to define the nature of the neurotoxicity (34). In the case of clinical deterioration or severe neurological symptoms, it is recommended to admit the patient and start prednisone 1–2 mg/kg orally or intravenously. For Guillain-Barré or myasthenia gravis-like syndromes, one should consider adding plasmapheresis or IVIG. These recommendations are based on level V Evidence (Studies without control group, case reports, expert opinions) and grade B level of recommendation (Strong or moderate evidence for efficacy but with a limited clinical benefit, generally recommended) (34).

## Neuro-ophthalmic immune-related adverse events

Neuro-ophthalmic immune-related adverse events (NOirAEs) encompass ocular inflammatory, systemic inflammatory, and systemic neurologic side effects that involve the visual and oculomotor pathways. In a recent systematic review, the overall incidence of NOirAEs is estimated at 0.46% (9). Overall, the most common reported irAEs are in association with pembrolizumab and nivolumab. Ipilimumab, the first ICI approved for cancer immunotherapy, carries a higher likelihood of irAEs but is less commonly used now except in combination therapy. It is associated with more side effects than anti-PD-1 or anti-PD-L1 agents, which is consistent with the overall rates of immune related adverse events in general (irAEs) (11, 19, 35). Nevertheless, the anti-PD-1 antibody pembrolizumab is associated with roughly 1/3 of NOirAE. Pembrolizumab and/or nivolumab have the most reported neuro-ophthalmic side effects overall (9, 20, 36). Average time from ICI initiation to onset of neuro-ophthalmic symptoms varies by dose and is on average at 2 cycles, or 1-3.5 months (9, 36). Due to the sheer variety of neuro-ophthalmic presentations, immune-related adverse events can be categorized into afferent versus efferent disorders, which broadly correlate with involvement of the central nervous system (CNS) versus peripheral nervous system (PNS).

### Afferent neuro-ophthalmic irAE (primarily CNS Disorders)

In the afferent visual pathway, ocular inflammation from uveitis is the most common form of OirAE, which can cause optic disc edema when posterior or intermediate. Optic disc edema is most commonly bilateral, and it is associated with ocular or orbital inflammation in about half of cases (36). There has also been a reported case of bilateral anterior uveitis with neuroretinitis (37). A case of visual field loss from ICI treatment complicated by melanoma-associated retinopathy has also been reported (38). Most of these cases have been managed, as with other forms of inflammatory uveitis, by using ocular or systemic corticosteroids as first-line treatment with an individualized approach.

Within the realm of optic neuropathy, inflammatory etiologies include retrobulbar demyelinating disease and optic disc edema from antibody-associated optic neuritis. Additionally, compressive optic neuropathy may result from inflammation of surrounding structures, and all of the above can result in optic nerve atrophy. Optic neuritis, particularly bilateral, is one of the most common NOirAE manifestations, 60% of cases having been associated with ipilimumab (9). Optic neuritis comprised 32% of the observed NOirAEs in a large retrospective case series (36). About half of these patients stopped ICI treatment. Nearly all patients experienced improved vision, disc edema, and optic neuritis post-treatment with steroids, although some cases required additional immunosuppression with IVIG and plasmapheresis, infliximab, and/or mycophenolate mofetil (9). In the context of malignancy, however, whether the ICI is continued or not depends on a careful balance between the morbidity risk of irAE therapy and the potential benefit and even life-sustaining control of malignancy.

Compressive optic neuropathy can result from orbital, apical, or pituitary inflammation, if severe enough. Orbital apex syndrome has been described from ipilimumab, which also caused hypophysitis (39). Hypophysitis is a rare form of inflammation of the anterior and posterior pituitary gland that causes a myriad of hormone deficiencies. Nevertheless, it has become widely recognized in anti-CTLA-4 antibody treatment and occurs in about 4% of patients on ipilimumab for metastatic melanoma (40). Hypophysitis can enlarge the pituitary gland, causing mass effect on the optic chiasm and nerves and resulting in bitemporal hemianopia (39).

Posterior to the chiasm, there is little in the literature regarding homonymous hemianopia from optic tract or radiation lesions. Nevertheless, bilateral visual disturbances and cortical blindness can occur in posterior reversible encephalopathy syndrome (PRES), which has been reported with use of ICIs (41, 42). Cognitive visual phenomena such as visual hallucinations also appear in reports of ICI-induced autoimmune encephalitis (43, 44). It is difficult to prove, however, that ICI cause cognitive visual phenomena in the absence of confirmatory neurologic signs of acute encephalitis, including positive neuroimaging, electro-encephalogram, or lumbar puncture.

In cases of afferent or CNS dysfunction, classification of diagnosis should include a degree of severity as measured by grade, association with other peripheral nervous system or systemic disease, and response to corticosteroid treatment and/or ICI cessation (if severe). Neuroimaging, including MRI brain and orbit with and without contrast, should be undertaken. Cerebrospinal fluid and serologic evaluation for infectious, neoplastic, or paraneoplastic etiology, is critical to ruling out other etiologies of suspected irAE, especially metastasis or non-ICI paraneoplastic etiology.

Paraneoplastic syndromes affecting the CNS can be novel autoimmune diseases triggered by ICI therapy; alternatively, they can be pre-existing paraneoplastic or autoimmune conditions that are recurrent after treatment or augmented by the ICI therapy (45–47). N-methyl-D-aspartate (NMDA) receptor, glial fibrillary acid protein (GFAP), antineuronal nuclear antibody type 1 (ANNA-1), collapsin response-mediator protein-5 (CRMP-5), and more antineuronal antibodies may be detected in higher number in CSF or serum of patients on ICI therapy than those without it (46). Neural antibodies such as Ma2 are found more often in patients on ICI therapy complicated by autoimmune encephalitis. They also require aggressive immunotherapy, permanent ICI cessation, and are associated with poor prognosis with high mortality (up to 50% in Ma2-associated limbic encephalitis patients) (46). Treatment and interdisciplinary collaborative needs will vary based on the degree and location of the damage. For example, with hypophysitis, co-management with endocrinology and possibly neurosurgery may be indicated.

### Efferent neuro-ophthalmic irAE (primarily PNS disorders)

ICIs are also responsible for efferent motor disorders, usually involving the peripheral nervous system. Mortality rates appear to be higher for efferent adverse events such as myasthenia gravis and myopathy/myositis cases than with afferent adverse events. The most severe and prevalent efferent NOirAE is immune related myasthenia gravis (irMG), comprising 45% of NOirAE in a comprehensive review (9, 48). In a recent review of 47 cases of irMG, nearly 30% of cases were fatal, versus 6-8% of classical MG (49). Fatigable ptosis and diplopia manifested in 79% of those patients, with purely ocular MG symptoms and signs in 15%. Most of the irMG adverse events were found in patients on anti-PD-1 antibodies, primarily nivolumab and pembrolizumab. Average time of onset was at one month, between cycles 2 and 3 of ICI administration. Respiratory failure and aspiration pneumonia were the most common causes of fatality. The presence of MG prior to ICI initiation increased the risk of getting irMG but not risk of fatality, and no cases had thymoma.

Immune-related myositis also causes efferent ocular motility dysfunction and may or may not be associated with irMG. Myositis is diagnosed based on the presence of elevated serum creatine kinase levels, muscle ultrasound, diaphragm nerve conduction study, electromyography, single muscle electromyography, and even muscle biopsy when required (49). Orbital inflammatory disease often presents concurrently with ocular motility dysfunction and myositis, and in some settings may threaten vision with compressive optic neuropathy (24, 50, 51). A recent study found orbital and extraocular muscle inflammation in 22.6% of NOirAE cases, all of whom presented with diplopia (36). Medications implicated are pembrolizumab and ipilimumab with or without nivolumab. Unlike thyroid-associated orbital inflammation, ICI-related myositis includes tendon sheaths and likely requires more substantial immunosuppression than corticosteroids (9).

Cranial neuropathy occurs as a side effect of ICI therapy as well. Associated cranial nerves (CN) are most commonly facial nerve, vestibulocochlear, and all three extraocular CN, but especially the abducens nerve. In a recent case series also involving the CN2 optic nerve, 30% of patients had persistent deficits after treatment, including hearing difficulty or vision loss (24, 36, 52). Most CN palsies present early during treatment, on average at 1 month (24).

Acute immune-related demyelinating polyneuropathies such as Miller-Fisher syndrome have more rarely been reported after use of ICIs (53, 54). Miller-Fisher syndrome classically with a combination of ophthalmoplegia, areflexia, and ataxia, and early treatment with steroid and IVIG have been advocated to decrease morbidity and mortality (53).

In the peripheral nervous system, classification of diagnosis should include a grade of severity, association with other CNS or systemic disease, and response to corticosteroid treatment and/or ICI cessation (if severe). Again, treatment differs by pathology. Cases of irMG were treated with steroids, IVIG, plasmapheresis, and even rituximab (48, 49, 55). In contrast, cranial neuropathies, even those associated with facial nerve palsy or leptomeningeal enhancement on MRI, mostly improved with oral or IV corticosteroid treatment, and ICI cessation was rarely required (24).

CNS disorders causing efferent dysfunction have been rare case reports and include internuclear ophthalmoplegia and opsoclonus-myoclonus-ataxia syndrome, as well as skew deviation (9, 24). Brainstem and cerebellar encephalitis can cause nystagmus and may be associated with several anti-neuronal antibodies as part of paraneoplastic syndromes seen in ICI therapy. Paraneoplastic antibody-associated syndromes have been found more commonly with CNS than PNS disorders (46).

### Neuro-ophthalmic irAE from systemic disease

Systemic disease from ICI therapy includes severe neuro-ophthalmic manifestations, most notably the rheumatologic and endocrine systems. Giant cell arteritis (GCA) and polymyalgia rheumatica-like irAE have been reported causes of vasculitis in association with PD-L1 inhibitors (9, 19, 36, 56, 57). Cases were diagnosed similarly to idiopathic GCA and treated with high-dose steroids. Interestingly, such cases have incidentally added to the understanding of GCA pathophysiology itself and have led to additional investigations in this field (58–61). For example, gene expression profiling of arteries showed a high expression of PD-L1 in dendritic cells of healthy arteries. Conversely, arteries affected by GCA showed low to no coinhibitory PD-L1 expression, but high co-stimulatory CD80 (B7-1) and CD86 (B7-2) expression, a finding which provides a potential mechanism for unopposed T-cell activation. Furthermore, T cells within granulomas of GCA temporal arteries were PD-1 positive (60). These findings, in concert with side effects associated with vasculitis in patients on ICI therapy, suggest that immune mediators may play a major role in GCA pathogenesis and can potentially identify targets for treatment of GCA itself.

Endocrine disorders due to ICI therapy have been described and include thyroid and pituitary disorders. Hypophysitis can present with vague symptoms such as headache, and it may lead to severe adverse events such as Addison crisis and/or diabetes insipidus (62). Thyroid ICI-related irAE in melanoma manifests as either destructive thyroiditis starting with a transient hyperthyroid phase and followed by permanent hypothyroidism, or as autoimmune hyperthyroidism secondary to Graves disease with varying levels of extraocular muscle involvement (63). There is a reported 2 to 4-fold increased incidence of thyroid irAE with combination versus mono-therapy (64). The American Society of Clinical Oncology (ASCO) recommends standard Graves disease therapy for secondary hyperthyroidism from ICIs (65). In contrast, destructive thyroiditis is deemed self-limiting. In the hyperthyroid phase, ASCO recommends beta-blockers for symptom control and free T4 testing every 2 weeks. For the resulting hypothyroid state, thyroid replacement therapy with levothyroxine is used, whereby dose is adjusted for age and known cardiovascular disease. Repeat TSH and free T4 testing is recommended after 6 to 8 weeks, and the thyroid hormone dose is adjusted accordingly. Regarding cessation of ICI therapy, ASCO guidelines recommend considering a re-challenge of ICI when hyperthyroidism resolves to grade 2 or better. In contrast, ICI hold is recommended in hypophysitis at grade 2 irAE until work up is complete and appropriate hormone replacement is started. Taken together, the endocrine disorders all require acute and possibly chronic immunosuppression with corticosteroids, but they may be appropriately managed if identified and treated promptly (62, 64, 66, 67).

## Limitations of knowledge about neuro-ophthalmic immune related adverse events

While there is exponential growth in the reporting of irAEs, most are from individual case reports and reviews of those case reports. Big database searches such as those in IRIS registry or the FDA’s FAERS database are limited by reporting bias (23, 24).

Actual prevalence of NOirAEs is not known, despite several large retrospective studies. The only study published to date that provided population-based epidemiologic data for NOirAE used the Kaiser electronic medical record search to report 1-year irAEs for uveitis and non-uveitis (scleritis, papilledema, optic neuritis, optic atrophy, cranial nerve 3, 4, or 6 mononeuropathy, internuclear ophthalmoplegia, and myasthenia gravis) (21). This study added valuable information regarding risk of uveitis and NOirAEs in patients with a history of uveitis and melanoma versus non-melanoma cancers involving a large and diverse population sample from one region of the United States. The use of ICIs in this population increased risk of OirAE in patients with a history of uveitis, especially in melanoma treatment (22). Nevertheless, prospective data collection with specific unified outcome measures will be needed to discover what really determines the success of treatment for and prognosis of these irAEs.

Another of the major diagnostic limitations is the difficulty in determining whether NOirAEs are due to the treatment itself or due to the original malignancy. Clues to ICI toxicity involve timing of the neurologic signs or symptoms, in which the AE often occurs 1-3 months after initiation of treatment. Other clues are the presence of pre-existing autoimmune conditions, presence of concurrent systemic irAEs, improvement with ICI cessation and/or corticosteroid administration, and the presence of known neurologic autoantibodies such as acetylcholine receptor (AchR) antibody-associated myasthenia gravis (33). Whether a paraneoplastic syndrome can be differentiated from an ICI adverse event is uncertain, but ICI therapy can trigger paraneoplastic syndromes, and the two occur in higher frequency together than apart (45).

Additionally, while there is retrospective data associating history of uveitis with high recurrence rates (21), we do not yet know all the risk factors that predispose patients to NOirAEs, nor which patients will respond favorably to one or another agent taken to manage the side effects. One study pointed to higher BMI and repeat treatment with higher rates of irAEs from pembrolizumab (68). Comprehensive risk factor determination will depend on larger, more centralized, and prospective database analysis.

Management of adverse events is based largely on management of similar autoimmune diseases, but prospective and comprehensive data is lacking regarding treatment medication choice (corticosteroids or otherwise), including superiority of dose and route. It is unknown if optimal treatment should depend on patient-specific biologic factors, or which type of treatment may work for a specific NOirAE. It is also unknown how combination treatment affects NOirAE incidence and severity, although combined anti-CTLA-4 and anti-PD-1 have been associated with a higher frequency of ophthalmoplegia relative to other adverse events (69).

There are also remaining questions regarding dose-dependent response to therapies, either by one or multiple combination agents. According to one retrospective study, CTLA-4 inhibitor monotherapy exhibited a dose/exposure dependence on most AE types evaluated, which was not present with PD-1 inhibitor monotherapy (70). Further characterizations of these potential relationships are lacking.

## Future directions

### Future of ICI immunotherapies

From the evidence available, it is clear that neuro-ophthalmic complications are associated with ICI agents, both with CTLA-4 and PD-1/PD-L1 inhibitors. These agents, however, are in evolution. New ICIs and other co-inhibitory receptors are under investigation for future therapeutic targets. Some of these targets are immune checkpoint inhibitors, directly affecting T cell function: Tim-3 (T-cell immunoglobulin-3, which promotes T cell exhaustion), Lag-3 (lymphocyte activation gene-3, which binds to MHCII and causes downregulation of cytokines, CD4 and CD8 T cells, and T regulatory phenotype adoption), and B7-H3 molecules (which bind to CD28 co-inhibitory pathways and dampen T cell activation, proliferation, and cytokine production) (10). Some are inhibitory targets beyond immune checkpoints, indirectly affecting T cell function or pathways, such as the Carcinoembryonic antigen-related cell adhesion molecules (CEACAM) family of proteins (which mediate different physiological effects ranging from tissue organization and angiogenesis to immune modulation), CCL2/CCR2 (chemokines, which are used by cancer cells to recruit immunosuppressive cells, promote angiogenesis, and facilitate tumor growth and differentiation), and CD47 (a marker of self-recognition that prompts an anti-phagocytic signal) (1, 10). This list continues to expand due to growing identification of cellular targets for monoclonal antibodies, and it will continue to grow. It will be important to make room for these new therapeutic targets to manage future side effects as well as therapeutic alternatives, as the pace of innovation in monoclonal antibody therapy accelerates.

### Non-ICI related immune adverse events

Non-ICI related immune adverse effects can also be seen in other targeted molecular antibody therapies such as B-Raf proto-oncogene serine/threonine kinase gene (BRAF) and mitogen/extracellular signal-regulated kinase (MEK) antibodies. These molecules mediate alternate pathways through distinct mechanisms involving downstream molecular signaling in patients with specific mutations thought causative of malignancy in colorectal cancer and melanoma (10, 71, 72). OirAE include uveitis, macular edema, and choroiditis (72, 73). A recent single institution retrospective case series of 901 patients on anti-BRAF therapy reported a 1.6% prevalence of ophthalmic side effects, including one patient with cranial nerve 6 palsy, in which treatment allowed for continuation of BRAF inhibitors (74).

### Developing a standardized classification system for NOirAE

Due to multi-system involvement manifesting as side effects within both the afferent and efferent realms, and the diversity of reactions in almost every part of the body, neuro-ophthalmic irAEs are often difficult to contain within one anatomical framework. In addition, new observations are coming into the literature frequently. Therefore, we propose categorizing the NOirAEs into an anatomical framework that is categorized by afferent visual pathways within the central nervous system, efferent oculmotor pathways within the peripheral nervous system, efferent manifestations of central nervous system disease, and systemic manifestations (see **Table 2**). Like the proposed classification system in neurologic irAEs, categorizations of disease subtype, diagnostic certainty, severity, autoantibody association, exacerbation of pre-existing disease or *de novo* presentation, and presence or absence of concurrent systemic irAE, should be collected prospectively.

**Table 2:** Proposed anatomical classification of neuro-ophthalmic immune-related adverse events.

Pathway	Anatomic site	Example
**Afferent visual pathway/CNS**	Intraocular optic nerve pathology	Optic disc edema secondary to uveitis
	Pre-chiasmal Optic nerve	Optic Neuritis, orbital compressive optic neuropathy
	Chiasm	Hypophysitis with compressive optic neuropathy*
	Post-chiasmal	Cortical blindness secondary to parieto-occipital lobe involvement from PRES
	Cognitive	Hallucinations secondary to autoimmune encephalitis
**Efferent oculomotor pathway/PNS**	Neuromuscular junction	Ocular myasthenia gravis
	Orbit	Orbital myositis Orbital Inflammatory Disease
	Cavernous Sinus	Tolosa-Hunt syndrome**
	Cranial Nerve	Cranial Nerve III palsy, Miller-Fisher syndrome
**Efferent oculomotor pathway/CNS**	Brainstem	Internuclear ophthalmoplegia
**Systemic/Other**	Vascular	Giant Cell Arteritis
	Endocrine	Graves Disease, thyroiditis

CNS, central nervous system; PNS, peripheral nervous system; PRES, posterior reversible encephalopathy syndrome.

*Hypophysitis has been reported but no reports yet found with chiasmal compression.

**No reports yet found.

### Developing a centralized NOirAE living database

The neuro-ophthalmic community would benefit greatly from developing standard definitions of NOirAEs by grade, which could guide treatment by severity (see examples in **Table 1**). With improved standards of practice, prognosis determination would be more accurate. To do this, however, evidence base must improve and, if possible, include both a retrospective streamlined database as well as prospective data collection standards during future clinical trials. There is a developing precedent in oncology, as evidenced by work presented at the 2020 ASCO Annual Meeting: “Alliance A151804, a national biorepository to advance studies of immune-related adverse events.” (75) This study provides the National Clinical Trial Network with a centralized repository for biospecimens to be used in translational studies of molecular pathogenesis and treatment of irAEs.

Whether biospecimen collection itself is necessary or not in NOirAE collection, we propose a centralized adverse event database that would compile data from all treatment centers regarding NOirAEs. Information collected should include type and dosing schedule of treatment (both for the etiology of NOirAE and its management), a comprehensive collection of all known ICI and other monoclonal antibody treatment, radiotherapy or other chemotherapeutic combinations, and offer quick communication between neuro-ophthalmologists and oncologists who are actively managing cases in interdisciplinary settings. Such a database would be dynamic and collect the most recent data from both post-market drug use as well as prospective clinical trial data to provide a living document that evolves as the drug market evolves. It would also provide a database to study whether treatment of cancer is successful despite the NOirAE, to study how NOirAE treatment affects cancer prognosis overall, and to provide a framework for management guidelines going forward.

### Developing standards of care for NOirAE

Using collective databases for irAE, ASCO and ESMO provide standard treatment recommendations based on type of irAE and severity grade (34, 65). For example, regarding the irAE hyperthyroidism, asymptomatic grade 1 findings require no intervention, but grade 2 and above require thyroid suppression medication, and grade 3 and 4, hospitalization and cessation of ICI. With a standardized NOirAE database to analyze, neuro-ophthalmology and oncology would be able to develop similar recommendations for NOirAEs. This would result in irAE management that is optimized for severity, standardized over sites, and easily accessible by providers with the greatest population burden.

We have much to learn, and neuro-ophthalmologists have a responsibility to maintain awareness of and look for potential NOirAEs, to establish the correct diagnosis, and to manage patients effectively. In conclusion, there is much work to do and establishing a multicenter, prospective event database will help us answer these remaining questions so important to patient care.

## Author contributions

KW outlined, drafted, and wrote the article, tables, and figure. LG conceptualized, edited, and provided extensive feedback. All authors contributed to the article and approved the submitted version.

## Conflict of interest

The authors declare that the research was conducted in the absence of any commercial or financial relationships that could be construed as a potential conflict of interest.

## Publisher’s note

All claims expressed in this article are solely those of the authors and do not necessarily represent those of their affiliated organizations, or those of the publisher, the editors and the reviewers. Any product that may be evaluated in this article, or claim that may be made by its manufacturer, is not guaranteed or endorsed by the publisher.
